
JOURNAL INFORMATION
==============================
NLM Title Abbreviation: Microb Biotechnol
Journal ID: Microb Biotechnol
NLM Journal ID: 101316335
Journal ID: mbt2

Microbial Biotechnology

EISSN: 1751-7915
Publisher: Wiley

ARTICLE INFORMATION
==============================
PMCID: PMC3896942
DOI: 10.1111/1751-7915.12082
Article version: 1
Subjects: Issue Information

Issue information

Print publication date: 2014 Jan
Electronic publication date: 2013 Dec 10
Volume: 7
Issue: 1
First page: i
Last page: iii
Copyright: © 2013 The Authors. Microbial Biotechnology published by John Wiley & Sons Ltd and Society for Applied Microbiology
Copyright year: 2013
License: Re-use of this article is permitted in accordance with the Creative Commons Deed, Attribution 2.5, which does not permit commercial exploitation.
License URL: https://creativecommons.org/licenses/by/2.5/

==============================
VOLUME 7 NUMBER 1 JANUARY 2014 www.microbialbiotech.com ISSN 1751-7907

### microbial biotechnology

Open Access

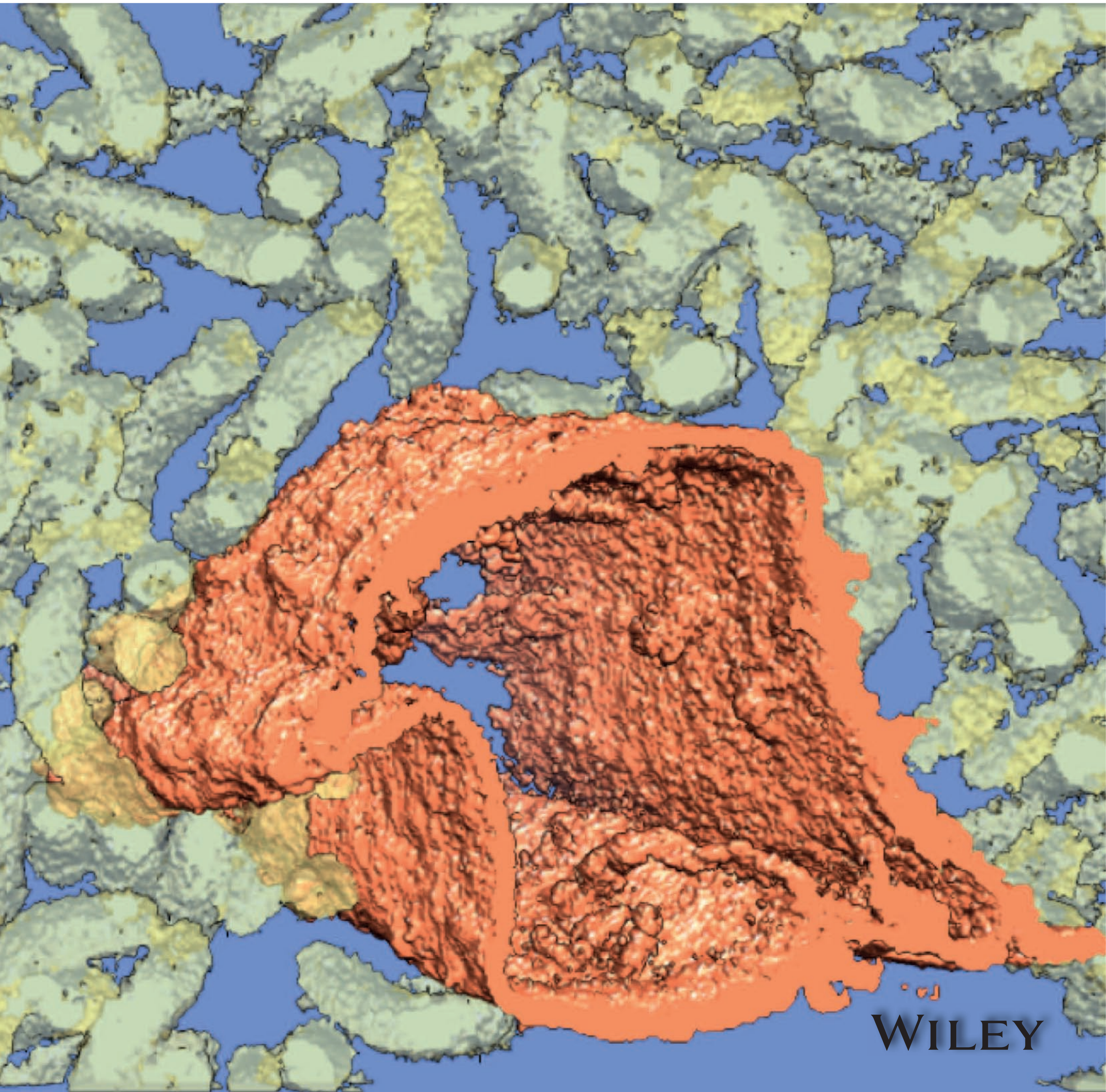

WILEY

### microbial biotechnology

Open Access

### EDITORS

**Kenneth N. Timmis** (Editor-in-Chief), Institute of Microbiology, Technical University, Braunschweig, Spielmannstr. 7, 38106 Braunschweig, Germany.
Tel.: +49 531 6181 4000;
Fax: +49 531 6181 4199;
e-mail: kti@helmholtz-hzi.de

**Juan L. Ramos**, Estación Experimental del Zaidín – CSIC, C/ Prof. Albareda, 1, E-18008 Granada, Spain.
Tel.: +34 958 181 608;
Fax: +34 958 135 740;
e-mail: jlramos@eez.csic.es

**Willem M. de Vos**, Laboratory of Microbiology, Wageningen University, Dreijenplein 10
Building 316, 6703 HB Wageningen, The Netherlands.
Tel.: +31 317 483 100;
Fax: +31 317 483 829;
e-mail: willem.deVos@wur.nl

**Willy Verstraete**, Laboratory of Microbial Ecology and Technology (LabMET), Ghent University, Coupure Links 653, B-9000 Ghent, Belgium.
Tel.: +32 9264 5976;
Fax: +32 9264 6248;
e-mail: willy.verstraete@ugent.be

**Martin Rosenberg**, Research & Development, Promega Corporation, 2800 Woods Hollow Road, Madison, WI 53711-5399, USA.
e-mail: martin.rosenberg@promega.com

### REVIEWS AND SPECIAL ISSUE EDITOR

**Juan L. Ramos**, Estación Experimental del Zaidín, Granada, Spain

### WEB ALERTS

**Larry Wackett**, University of Minnesota, St Paul, USA

### EDITORIAL BOARD

Lars Angenent, USA
Steven A. Banwart, UK
Francisco Barona-Gómez, Mexico
Shimshon Belkin, Israel
Giovanni Bertoni, Italy
Ian Booth, UK
Uwe Bornscheuer, Germany
Michael Bott, Germany
Harald Brussow, Switzerland
Rita Colwell, USA
Milton Costa, Portugal
Jiri Damborsky, Czech Replublic
Craig Daniels, Canada
Eduardo Diaz, Spain
Gerrit Eggink, Germany
Thomas Egli, Switzerland
Romilio Espejo, Chile
Manuel Ferrer, Spain
Julia Foght, Canada
Jim K Fredrickson, USA
Michael Galperin, USA
Jose Luis Garcia, Spain
Harry Gilbert, UK
Bernardo Gonzalez, Chile
Guido Grandi, Italy
Miguel G. Guerrero, Spain
Carlos A. Guzman, Germany
Ken Hammel, USA
Bernhard Hauer, Germany
Terry C. Hazen, USA
Michael Hecker, Germany
Hermann J. Heipieper, Germany
Monica Höfte, Belgium
Karl-Erich Jaeger, Germany
Dieter Jahn, Germany
Junichi Kato, Japan
Staffan Kjelleberg, Australia
Todd R. Klaenhammer, USA
Alexander M. Klibanov, USA
Rob Knight, USA
Tino Krell, Spain
Oscar Kuipers, The Netherlands
Robert Larossa, USA
Sang Yup Lee, Republic of Korea
Daniel van der Lelie, USA
Steven Lindow, USA
Mark van Loosdrecht, The Netherlands
Victor de Lorenzo, Spain
José M. Luengo, Spain
Ben Lugtenberg, The Netherlands
Mohamed A. Marahiel, Germany
Angel T. Martínez, Spain
Michael McInerney, USA
Rainer U. Meckenstock, Germany
Jan Roelof van der Meer, Switzerland
Diego de Mendoza, Argentina
Gabriella Molinari, Germany
Jens Nielsen, Sweden
Per Halkjær Nielsen, Denmark
Fergal O'Gara, Ireland
Sven Panke, Switzerland
David Payne, USA
Miguel A. Peñalva, Spain
Dietmar Pieper, Germany
Korneel Rabaey, Belgium
Jeroen Raes, Belgium
Lutgarde Raskin, USA
Fernando Rojo, Spain
Wilfred F.M. Röling, The Netherlands
Eliora Z. Ron, Israel
Sima Sariaslani, USA
Uwe Sauer, Switzerland
Bernhard Schink, Germany
Claudia Schmidt-Dannert, USA
Anna Schnürer, Sweden
Ana Segura, Spain microbial biotechnology

Open Access

**EDITORIAL BOARD cont.**

Roland J. Siezen, The Netherlands
Douwe van Sinderen, Ireland
Detmer Sipkema, The Netherlands
Hauke Smidt, The Netherlands
Gloria Soberón Chávez, México
Alexander Steinbüchel, Germany
Bas Teusink, The Netherlands
Svein Valla, Norway
Erick Vandamme, Belgium
Jos Vanderleyden, Belgium
L. A. M. van der Wielen, The Netherlands
Rene H. Wijffels, The Netherlands
Roland Wohlgemuth, Switzerland
Thomas K. Wood, USA
Gary Woodnutt, USA
Michail Yakimov, Italy
Liping Zhao, China

### SCOPE

*Microbial Biotechnology* publishes papers of original research reporting significant advances in any aspect of microbial applications, including, but not limited to biotechnologies related to:

- microbial communities: structure:function relationships and communal behavior
- Green chemistry
- Primary metabolites
- Food, beverages and supplements
- Secondary metabolites and natural products
- Pharmaceuticals
- Diagnostics
- Agriculture
- Bioenergy
- Biomining, including oil recovery and processing
- Bioremediation
- Biopolymers, biomaterials
- Bionanotechnology
- Biosurfactants and bioemulsifiers
- Compatible solutes and bioprotectants
- Biosensors, monitoring systems, quantitative microbial risk assessment
- Technology development
- Protein engineering
- Functional genomics
- Metabolic engineering
- Metabolic design
- Systems analysis, modelling
- Process engineering
- Biologically-based analytical methods
- Microbially based strategies in public health
- Microbially based strategies to influence global processes

***Open Access and Copyright***. All articles published by *Microbial Biotechnology* are fully open access: immediately freely available to read, download and share. All *Microbial Biotechnology* articles are published under the terms of the Creative Commons Attribution License which permits use, distribution and reproduction in any medium, provided the original work is properly cited, and allows the commercial use of published articles.

Copyright on any research article in a journal published by *Microbial Biotechnology* is retained by the author(s). Authors grant Wiley a license to publish the article and identify itself as the original publisher. Authors also grant any third party the right to use the article freely as long as its integrity is maintained and its original authors, citation details and publisher are identified. Further information about open access license and copyright can be found on http://www.wileyopenaccess.com/details/content/12f25db4c87/Copyright--License.html.

***Use by commercial 'for-profit' organisations***. Use of Wiley Open Access articles for commercial, promotional, or marketing purposes requires further explicit permission fromWiley (corporatesales@wiley.com) and will be subject to a fee. Commercial purposes include:

- Copying or downloading of articles, or linking to such articles for further redistribution, sale or licensing;
- Copying, downloading or posting by a site or service that incorporates advertising with such content;
- The inclusion or incorporation of article content in other works or services (other than normal quotations with an appropriate citation) that is then available for sale or licensing, for a fee (for example, a compilation produced for marketing purposes, inclusion in a salespack);
- Use of article content (other than normal quotations with appropriate citation) by forprofit organisations for promotional purposes;
- Linking to article content in e-mails redistributed for promotional, marketing or educational purposes;
- Use for the purposes of monetary reward by means of sale, resale, licence, loan, transfer or other form of commercial exploitation such as marketing products;
- Print reprints of Wiley Open Access articles can be purchased from corporatesales@wiley.com.

***Disclaimer***. The Publisher, Society for Applied Microbiology and Editors cannot be held responsible for errors or any consequences arising from the use of information contained in this journal; the views and opinions expressed do not necessarily reflect those of the Publisher, Society for Applied Microbiology and Editors; neither does the publication of advertisements constitute any endorsement by the Publisher, Society for Applied Microbiology and Editors of the products advertised.

Wiley Open Access articles posted to repositories or websites are without warranty from Wiley of any kind, either express or implied, including, but not limited to, warranties of merchantability, fitness for a particular purpose, or non-infringement. To the fullest extent permitted by law Wiley disclaims all liability for any loss or damage arising out of, or in connection with, the use of or inability to use the content.
